# Diagnostic performance of sonographic activity scores for adult terminal ileal Crohn’s disease compared to magnetic resonance and histological reference standards: experience from the METRIC trial

**DOI:** 10.1007/s00330-023-09958-6

**Published:** 2023-08-01

**Authors:** Shankar Kumar, Thomas Parry, Sue Mallett, Andrew Plumb, Gauraang Bhatnagar, Richard Beable, Margaret Betts, Gillian Duncan, Arun Gupta, Antony Higginson, Rachel Hyland, Roger Lapham, Uday Patel, James Pilcher, Andrew Slater, Damian Tolan, Ian Zealley, Steve Halligan, Stuart A. Taylor, METRIC study group

**Affiliations:** 1 Centre for Medical Imaging, University College London (UCL), 2nd Floor Charles Bell House, 43-45 Foley Street, W1W 7TS, London, UK; 2 Department of Radiology, Frimley Park Hospital, Surrey, UK; 3 Department of Radiology, Queen Alexandra Hospital, Portsmouth Hospitals NHS Trust, Portsmouth, UK; 4 Department of Radiology, Ninewells Hospital and Medical School, Dundee, UK; 5 Department of Radiology, Oxford University Hospitals NHS Foundation Trust, Oxford, UK; 6 Department of Radiology, St Mark’s Hospital, London North West University Healthcare NHS Trust, London, UK; 7 Department of Radiology, St James’ University Hospital, Leeds Teaching Hospitals NHS Trust, Leeds, UK; 8 Department of Radiology, St George’s University Hospitals NHS Foundation Trust, London, UK

**Keywords:** Crohn’s disease, Diagnostic imaging, Ultrasonography

## Abstract

**Objectives:**

The simple ultrasound activity score for Crohn’s disease (SUS-CD) and bowel ultrasound score (BUSS) are promising intestinal ultrasound (IUS) indices of CD, but studied mainly in small settings with few sonographers. We compared SUS-CD and BUSS against histological and magnetic resonance enterography (MRE) reference standards in a post hoc analysis of a prospective multicentre, multireader trial.

**Methods:**

Participants recruited to the METRIC trial (ISRCTN03982913) were studied, including those with available terminal ileal (TI) biopsies. Sensitivity and specificity of SUS-CD and BUSS for TI CD activity were calculated with 95% confidence intervals (CI), from the prospective observations of the original METRIC trial sonographers against the histological activity index (HAI) and the simplified magnetic resonance index of activity (sMARIA).

**Results:**

We included 284 patients (median 31.5 years, IQR 23–46) from 8 centres, who underwent IUS and MRE. Of these, 111 patients had available terminal ileal biopsies with HAI scoring. Against histology, sensitivity and specificity for active disease were 79% (95% CI 69–86%) and 50% (31–69%) for SUS-CD, and 66% (56–75%) and 68% (47–84%) for BUSS, respectively. Compared to sMARIA, the sensitivity and specificity for active CD were 81% (74–86%) and 75% (66–83%) for SUS-CD, and 68% (61–74%) and 85% (76–91%) for BUSS, respectively. The sensitivity of SUS-CD was significantly greater than that of BUSS against HAI and sMARIA (*p *< 0.001), but its specificity was significantly lower than of BUSS against the MRE reference standard (*p *= 0.003).

**Conclusions:**

Particularly when compared to MRE activity scoring, SUS-CD and BUSS are promising tools in a real-world clinical setting.

**Clinical relevance statement:**

When tested using data from a multicentre, multireader diagnostic accuracy trial, the simple ultrasound activity score for Crohn’s disease (SUS-CD) and bowel ultrasound score (BUSS) were clinically viable intestinal ultrasound indices that were reasonably sensitive and specific for terminal ileal Crohn’s disease, especially when compared to a magnetic resonance reference standard.

**Key Points:**

- *The simple ultrasound activity score for Crohn’s disease and bowel ultrasound score are promising intestinal ultrasound indices of Crohn’s disease but to date studied mainly in small settings with few sonographers.*
- *Compared to histology and the magnetic resonance reference standard in a multicentre, multireader setting, the sensitivity of simple ultrasound activity score for Crohn’s disease is significantly greater than that of bowel ultrasound score.*
- *The specificity of simple ultrasound activity score for Crohn’s disease was significantly lower than that of bowel ultrasound score compared to the magnetic resonance enterography reference standard. The specificity of both indices was numerically higher when the magnetic resonance enterography reference standard was adopted.*

**Supplementary information:**

The online version contains supplementary material available at 10.1007/s00330-023-09958-6.

## Introduction

Identifying and treating inflammatory activity in Crohn’s disease (CD) are fundamental to optimise management and reduce subsequent penetrating and stricturing disease [1]. Cross-sectional imaging including magnetic resonance enterography (MRE) and intestinal ultrasound (IUS) is used routinely to diagnose and monitor CD [2, 3], and they are viable alternatives to colonoscopy [4–7]. IUS has several advantages over MRE, being rapid in both bedside and outpatient settings, inexpensive, better tolerated by patients and avoiding contrast administration [8–12]. There has been considerable interest in developing and validating sonographic scores that quantify disease activity, in the hope that more systematic interpretation will improve consistency, aid comparison between consecutive examinations and facilitate response assessment. A range of US activity scores are proposed, including the simple ultrasound score for Crohn’s disease (SUS-CD) and bowel ultrasound score (BUSS), which have both been developed recently using robust methodology, and perform well against colonoscopy [13, 14].

To date, these promising indices have been derived and initially evaluated in single- or dual-centre studies using few, highly specialised sonographers, so their performance characteristics in generalised practice are unknown [13–16]. Whilst mucosal assessments with endoscopic or histological scoring are frequently used as a reference standard for disease activity, they can neglect transmural disease, something captured by cross-sectional imaging. Accordingly, we evaluated the accuracy of SUS-CD and BUSS to identify terminal ileal CD activity using both histopathological and magnetic resonance reference standards, obtained as part of a prospective, multicentre, multireader diagnostic accuracy trial [4].

## Materials and methods

### Study population and design

The MR Enterography or Ultrasound in Crohn’s disease (METRIC) trial (Current Controlled Trials ISRCTN03982913) was a prospective multicentre diagnostic accuracy study comparing MRE and IUS in adult CD [4, 17]. Patients with newly diagnosed or established CD suspected of relapse were recruited from eight UK National Health Service (NHS) hospitals, and underwent IUS and MRE, as well as any other investigations such as endoscopy, required for standard care. The SUS-CD and BUSS were described after the METRIC trial completion; thus, the present study is a post hoc analysis rather than a pre-specified secondary outcome of the METRIC trial.

**Fig. 1:**
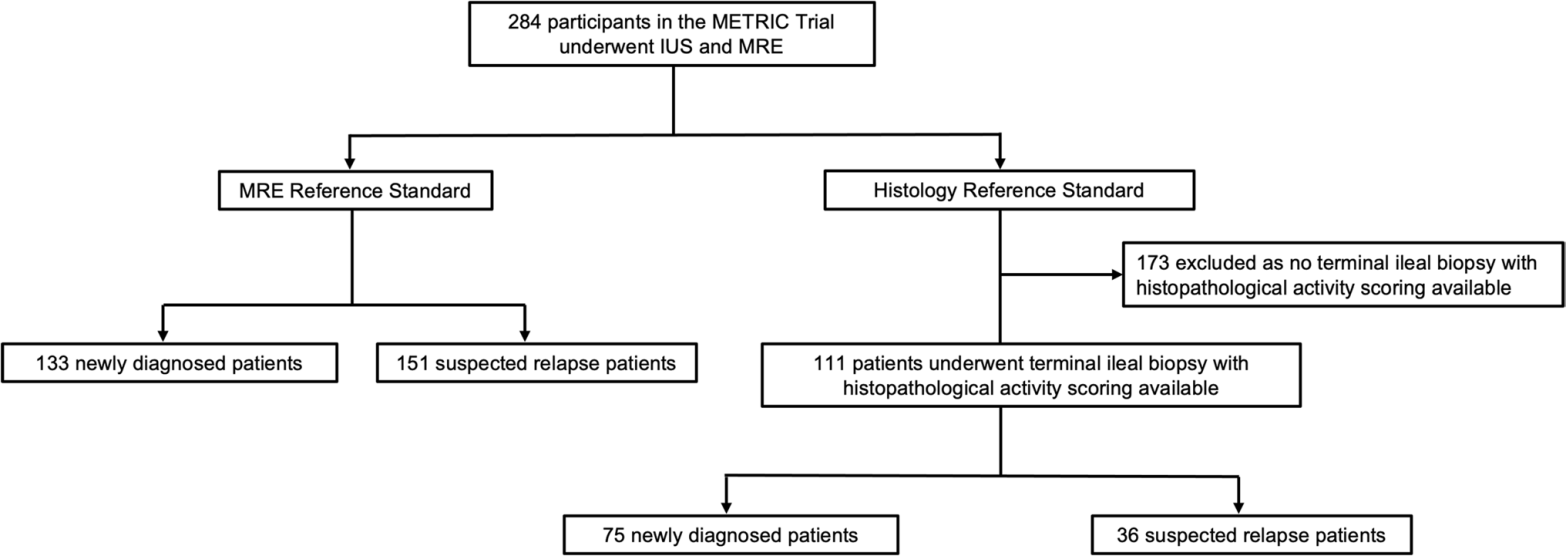
Flow chart of the study population

For the current study, we considered all patients (both newly diagnosed and suspected relapse) recruited to the METRIC trial for the comparison of IUS against a MRE standard of reference. This included some patients with a terminal ileal biopsy sample (with histological activity scoring), available within 4 weeks of IUS (Fig. 1), for the comparison of IUS against a histological standard of reference. This cohort has been described previously in a study investigating the diagnostic performance of MRE activity scores [18], but this prior report did not consider ultrasound activity scores (SUS-CD and BUSS), nor did it utilise an additional MRE reference standard. We reported results using the QUADAS-2 reporting guidelines for validation studies [19].

### IUS protocol

IUS was performed prospectively and according to local protocols, using standard imaging platforms, curvilinear (2 – 5 MHz) and linear (> 5 MHz) probes, without oral or intravenous contrast [4, 17]. Patients fasted for 4 h prior to the IUS study. The colour Doppler setting was 6–9 m/s. Radiologists performing IUS were Fellows of the Royal College of Radiologists, affiliated to the British Society of Gastrointestinal and Abdominal Radiology (BSGAR), and had a minimum of 1 year subspeciality training in gastrointestinal radiology. One sonographer performed IUS who had received formal training and performed IUS routinely in clinical practice. The operators performing IUS had a median of 8 years (IQR 4–11) of experience. All practitioners were blinded to clinical data, previous imaging studies and endoscopic findings, and prospectively completed a standardised clinical report form (CRF), documenting conventional IUS observations (Appendix 1). Data from these prospectively completed CRFs were used by the study coordinator (radiologist with 5 years experience of MRE and IUS) to retrospectively derive the SUS-CD and BUSS activity scores without re-reviewing images.

### Derivation of IUS activity scores

The SUS-CD and BUSS for the terminal ileum were calculated using pre-specified formulae from published methods [13, 14, 20], with the worst affected section assessed as follows:

$$\mathrm{SUS}-\mathrm{CD}=\mathrm{bowel}\;\mathrm{wall}\;\mathrm{thickness}+\mathrm{colour}\;\mathrm{Doppler}\;\mathrm{score}$$

$$\mathrm{BUSS}=0.75\times\mathrm{bowel}\;\mathrm{wall}\;\mathrm{thickness}\;+1.65\times\mathrm{bowel}\;\mathrm{wall}\;\mathrm{flow}$$

Full definitions for the activity scores are provided in Appendix 2.

**Fig. 2:**
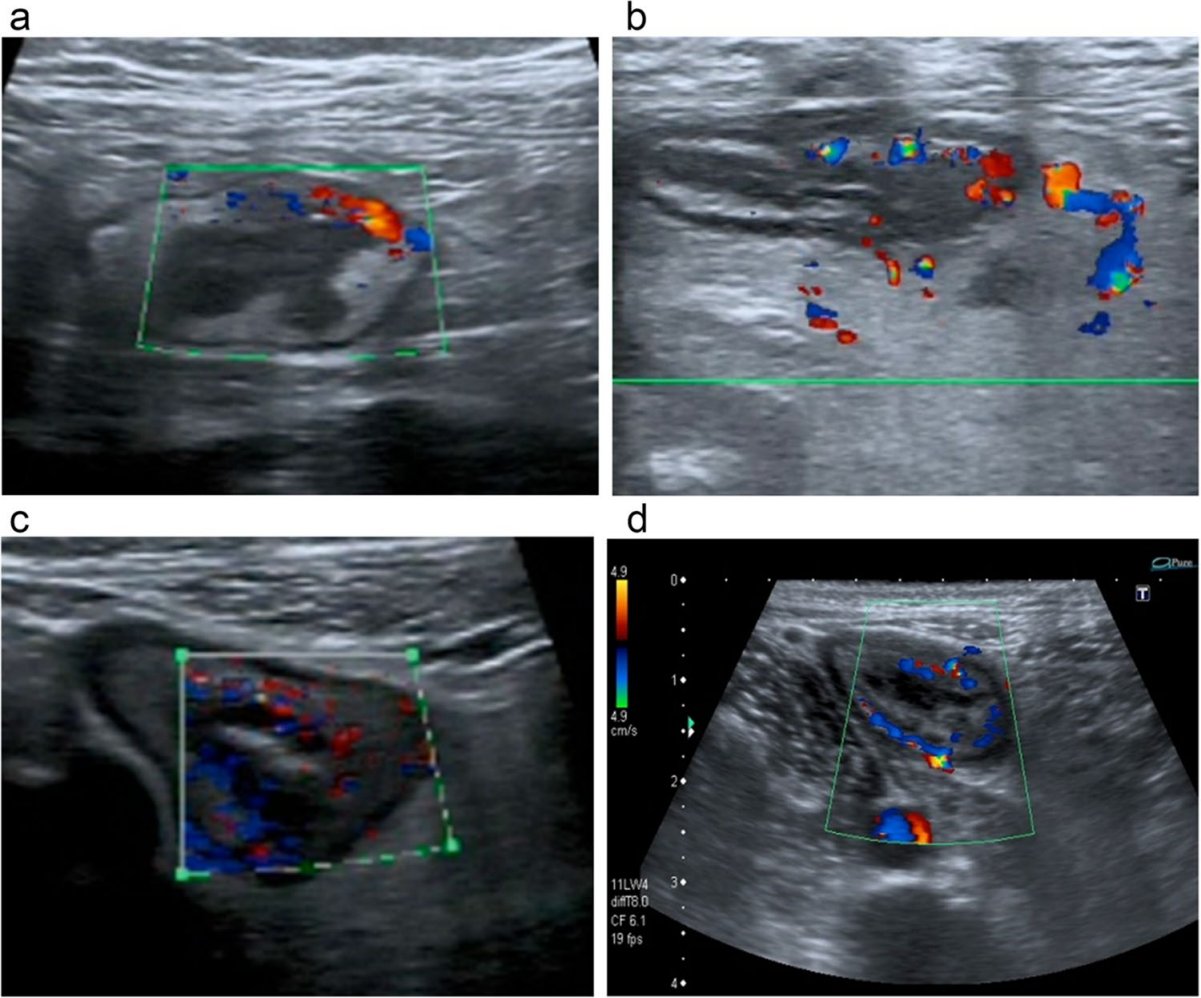
**a**, **b** Increased focal Doppler signal isolated to less than half the circumference on a trans-axial image compared to normal bowel in the same patient. **c**, **d** Increased generalised Doppler signal affecting more than half the circumference on a trans-axial image compared to normal bowel in the same patient

For the colour Doppler score component of SUS-CD, a priori we assigned a score of 1 to increased Doppler signal isolated to less than half the circumference on a trans-axial image when compared to an adjacent normal bowel loop in the same patient (Fig. 2a, b). Increased Doppler signal affecting more than half the circumference on a trans-axial image compared to normal bowel in the same patient was assigned a score of 2 (Fig. 2c, d). For BUSS, the bowel wall flow component scored 1, irrespective of whether the increased Doppler signal involved less or more than half the circumference compared to normal bowel, as per the original published article [14].

## Reference standards

### Histopathological reference standard

Terminal ileum biopsies were analysed by a specialist gastrointestinal histopathologist at each site, unaware of IUS findings. They scored the biopsy with the most severe inflammation according to the histological activity index (HAI) as follows: 0, remission; 1, mild activity; 2, moderate activity; 3, severe activity (Supplemental Table 1) [21].

### Magnetic resonance reference standard

The simplified magnetic resonance index of activity (sMARIA) is an MRE activity score that has been validated against endoscopic reference standards, and used in several studies [22–26]. We derived the terminal ileal sMARIA for all patients as described previously [18]. In brief, MRE was performed as per local protocols at each METRIC trial site using either 1.5- or 3-Tesla platforms, and a minimum number of sequences were acquired [4]. Radiologists were blinded to all patient data and prospectively completed a standardised CRF from which the sMARIA was derived. The score ranges from 0 to 5, and a value of 1 or more indicates active CD (Appendix 3) [22].

### Statistical analysis

The primary outcome was the difference in sensitivity and specificity between SUS-CD and BUSS for the activity of TI CD compared to the histological reference standard. We pre-specified active disease as HAI ≥ 1 [21]. The secondary outcome was the difference in sensitivity and specificity between SUS-CD and BUSS for the activity of TI CD relative to the MRE reference standard. We pre-specified active disease as sMARIA ≥ 1 [22]. We reported both outcomes stratified by newly diagnosed and suspected relapse patients. IUS activity score thresholds for active CD were taken from the published articles as ≥ 1 for SUS-CD [13] and > 3.52 for BUSS [14].

We calculated the sensitivity and specificity with Wilson’s 95% confidence intervals (CI) for each scoring system at the pre-specified thresholds. We used McNemar’s test to calculate the difference in sensitivity and specificity with exact 95% CI between SUS-CD and BUSS. All analyses were performed using Stata 17 (StataCorp). Statistical significance was based on 95% CI [27].

### Ethics

Ethical approval was granted for the original trial in September 2013 (13/SC/0394). All participants provided informed written consent including for research purposes [4, 17].

## Results

### Study population and patient characteristics

The METRIC trial recruited 284 participants from 8 institutions, all of whom underwent IUS and MRE. Of these, 133 (47%) were newly diagnosed and 151 (53%) had established CD (Fig. 1). Of the 111 patients who underwent colonoscopy and had terminal ileal histopathological activity scoring available, 75 (68%) were newly diagnosed and 36 (32%) had established CD [18]. IUS was performed by one of 19 practitioners. Patient demographics, clinical characteristics and HAI scores are presented in Table 1.

**Table 1:** Demographic and clinical characteristics of all patients

Characteristic	Newly diagnosed, *n *= 133	Suspected relapse, *n *= 151	All patients, *n *= 284
Age (years)	30 (21, 45)	33 (24, 46)	32 (23, 46)
Sex			
Male	69 (52)	61 (40)	139 (46)
Female	64 (48)	90 (60)	154 (54)
Medication			
ASA	30 (11)	16 (10)	46 (10)
Anti-TNF antibodies	16 (6)	5 (3)	21 (5)
Immunomodulator	54 (19)	24 (15)	78 (18)
Steroid	36 (13)	41 (26)	77 (17)
HBI	3 (1.5, 5)	5 (2, 67)	4 (2, 6)
EQ-5D	70 (50, 85)	67 (50, 80)	70 (50, 80)
CRP (mg/L)	9.1 (3.8, 24)	6 (2, 18)	7.7 (2.8, 21)
Calprotectin (μg/g)	425 (98, 600)	335 (86, 600)	341 (90, 600)
History of bowel surgery	11 (8)	72 (48)	83 (29)
US platform			
GE Healthcare	28 (21)	19 (13)	47 (17)
Philips	25 (19)	21 (14)	46 (16)
Siemens	28 (21)	42 (28)	70 (25)
Toshiba	52 (39)	69 (46)	121 (43)

All data are *n* (%) or median (inter-quartile range)

Abbreviations: *ASA* acetylsalicylic acid, *TNF* tumour necrosis factor, *HBI* Harvey-Bradshaw Index, *EQ-5D* EuroQol five-dimension questionnaire, *CRP* C-reactive protein, *US* ultrasound

Missing data: HBI = 9, EQ-5D = 12, CRP = 10, Calprotectin = 50

### Diagnostic accuracy of SUS-CD and BUSS for identifying active CD, using the histology reference standard

**Fig. 3:**
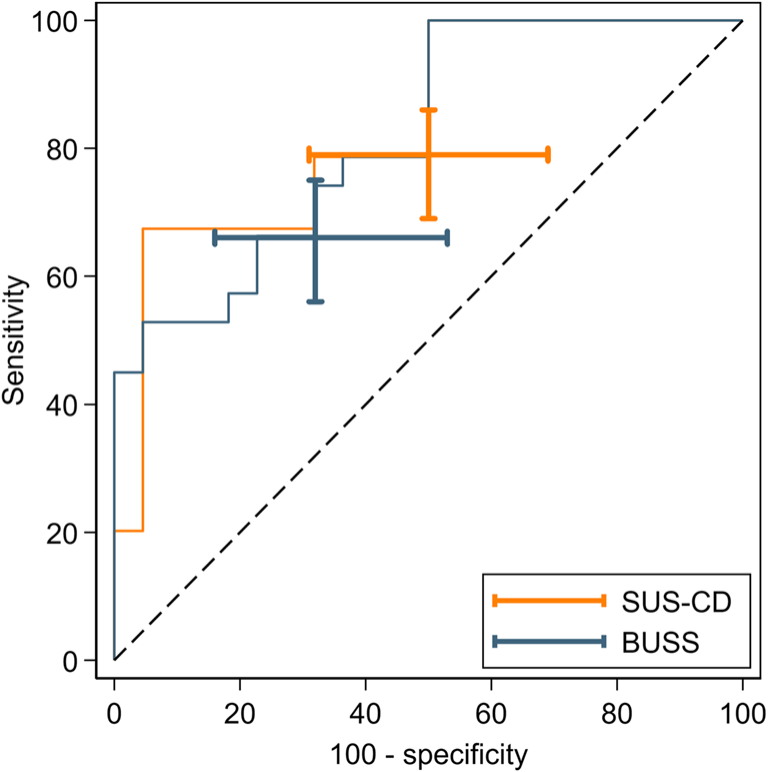
ROC curves of SUS-CD and BUSS for the activity of CD against the HAI reference standard. Capped I-beams represent 95% CI at the pre-specified thresholds

**Fig. 4:**
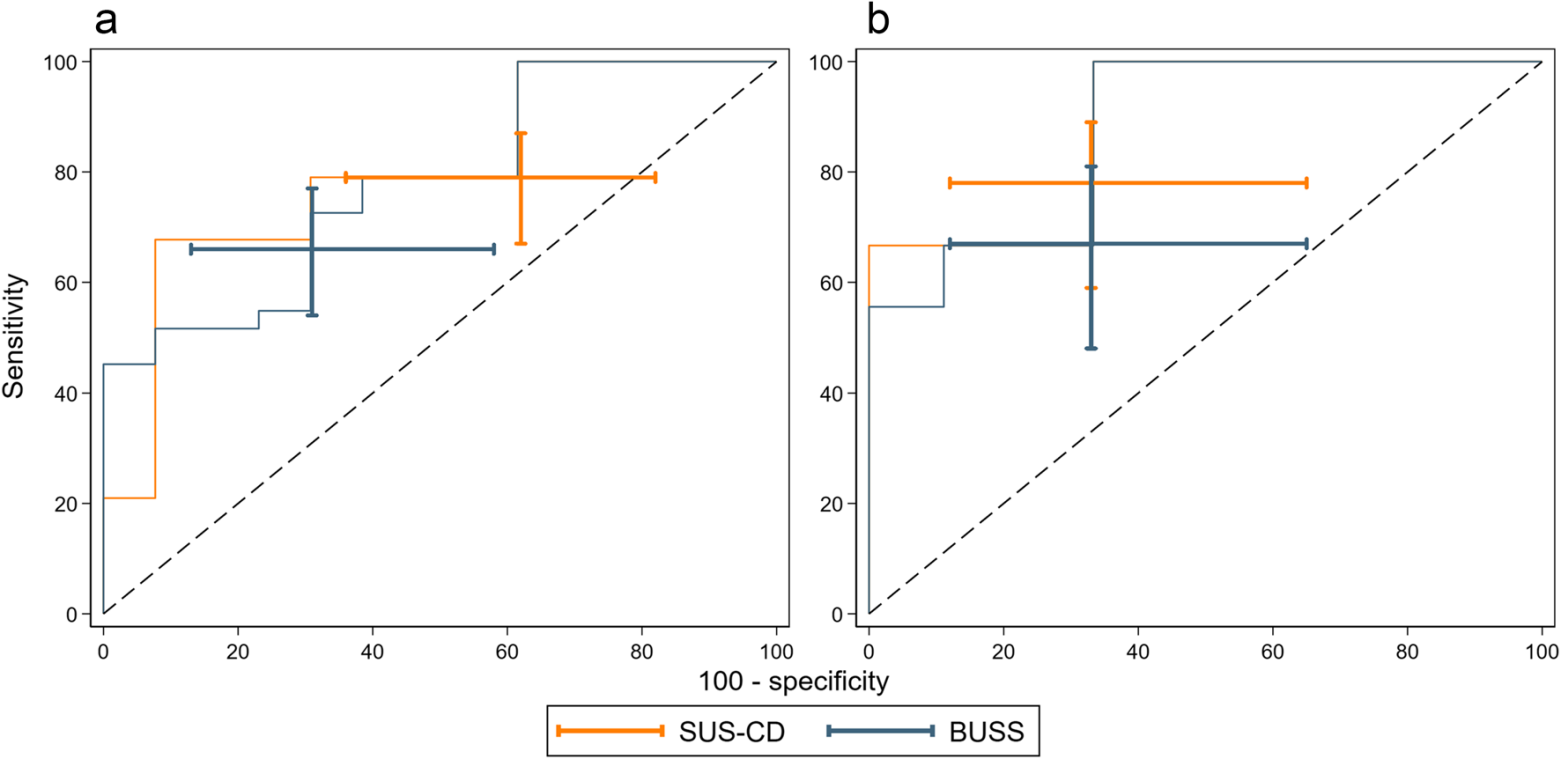
ROC curves of SUS-CD and BUSS for the activity of CD against the HAI reference standard, stratified by (**a**) newly diagnosed and (**b**) suspected relapse. Capped I-beams represent 95% CI at the pre-specified thresholds

Table 2 details the sensitivity and specificity of each IUS index for the activity of TI CD versus the histology reference standard. The corresponding ROC plots are presented in Figs. 3 and 4. The sensitivity of SUS-CD (79% [69, 86]) was significantly greater than that of BUSS (66% [56, 75]) with a difference of 12% (4, 20; *p *< 0.001). There was no significant difference in specificity (−18% [−39, 2]; *p *= 0.046).

**Table 2:** Diagnostic accuracy parameters of SUS-CD and BUSS scores for the activity of CD against the histology reference standard

	Sensitivity					Specificity					AUC	
	DP	SUS-CD	BUSS	Difference	*p*-value	DN	SUS-CD	BUSS	Difference	*p*-value	SUS-CD	BUSS
All patients	89	79 (69, 86)	66 (56, 75)	12 (4, 20)	< 0.001	22	50 (31, 69)	68 (47, 84)	−18 (−39, 2)	0.046	0.74 (0.64, 0.84)	0.76 (0.66, 0.85)
Newly diagnosed	62	79 (67, 87)	66 (54, 77)	13 (3, 23)	0.005	13	38 (18, 64)	69 (42, 87)	−31 (−64, 2)	0.046	0.71 (0.57, 0.85)	0.73 (0.60, 0.85)
Suspected relapse	27	78 (59, 89)	67 (48, 81)	11 (−4, 27)	0.083	9	67 (35, 88)	67 (35, 88)	0 (−11, 11)	1	0.78 (0.64, 0.93)	0.79 (0.65, 0.93)

Data are *n*, % (95% CI) or *p*-value

Abbreviations: *DP* disease positive, *DN* disease negative, *SUS-CD* simple ultrasound score for Crohn’s disease, *BUSS* bowel ultrasound sonographic score, *HAI* histological activity index

For the newly diagnosed group, the sensitivity of SUS-CD was significantly greater than that of BUSS, a difference of 13% (3, 23; *p *= 0.005). Again, there was no significant difference in specificity (−31% [−64, 2]; *p *= 0.046).

For the suspected relapse group, there was no significant difference in sensitivity or specificity between SUS-CD and BUSS.

By way of illustration, in 1000 hypothetical patients, SUS-CD would identify 631 true positives, 99 false positives, 171 false negatives and 99 true negatives. BUSS would identify 532 true positives, 63 false positives, 270 false negatives and 135 true negatives.

### Diagnostic accuracy of SUS-CD and BUSS for identifying active CD, using the magnetic resonance reference standard

**Fig. 5:**
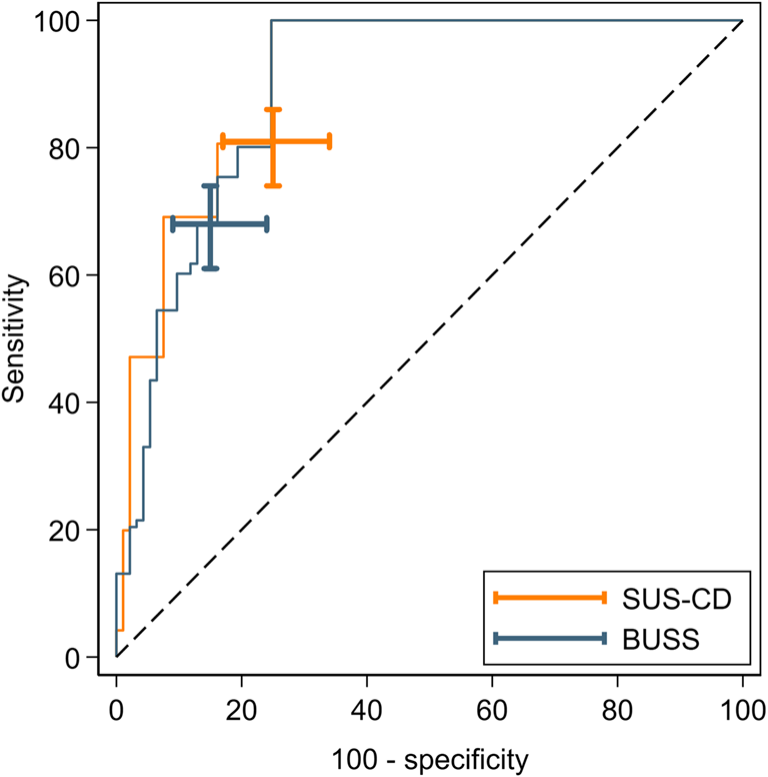
ROC curves of SUS-CD and BUSS for the activity of CD against the sMARIA reference standard. Capped I-beams represent 95% CI at the pre-specified thresholds

**Fig. 6:**
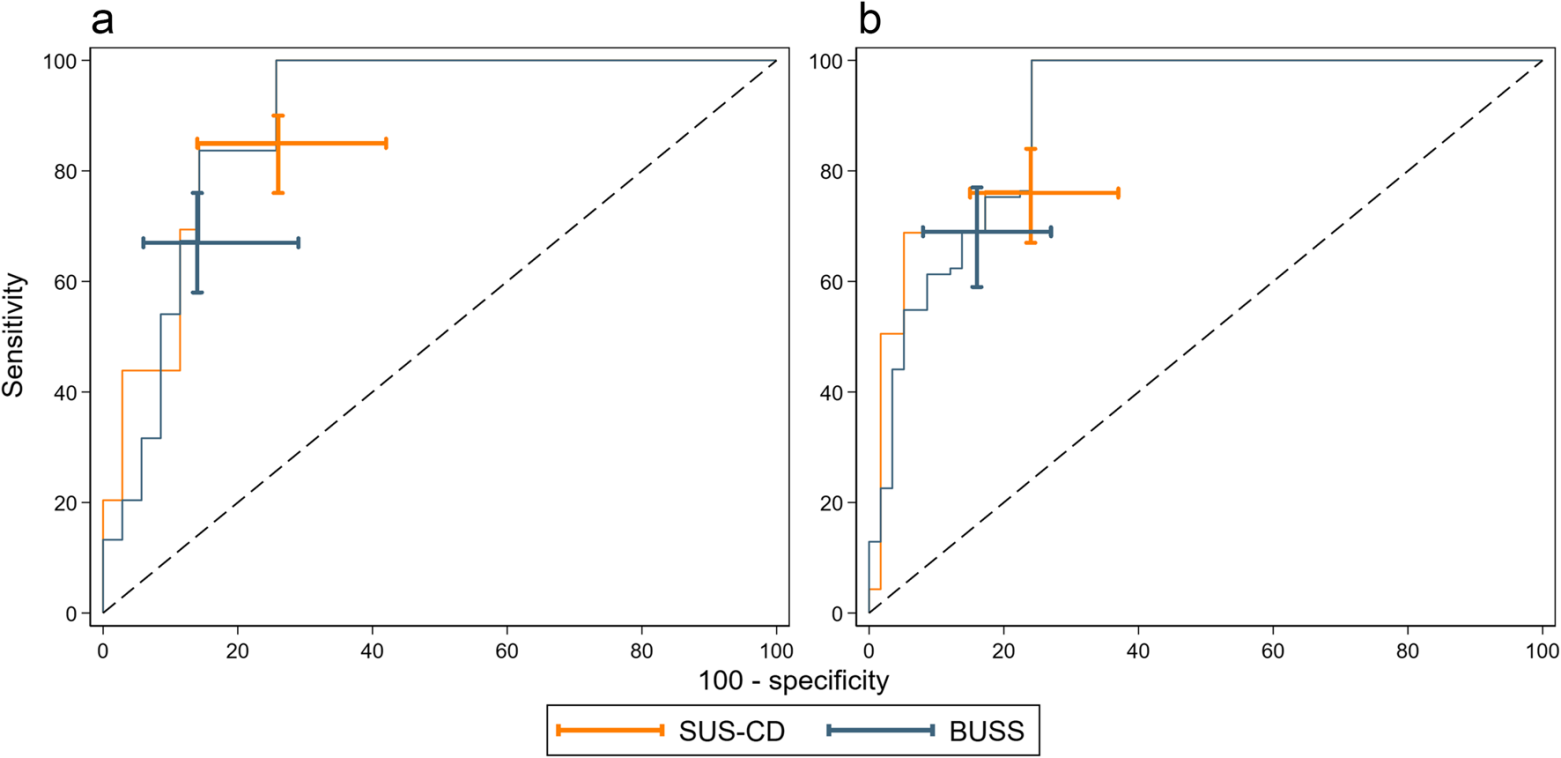
ROC curves of SUS-CD and BUSS for the activity of CD against the sMARIA reference standard, stratified by (**a**) newly diagnosed and (**b**) suspected relapse. Capped I-beams represent 95% CI at the pre-specified thresholds

The sensitivity and specificity of SUS-CD and BUSS compared to the MRE reference standard are presented in Table 3, with the corresponding ROC plots in Figs. 5 and 6.

**Table 3:** Diagnostic accuracy parameters of SUS-CD and BUSS scores for the activity of CD against the MRE reference standard

	Sensitivity					Specificity					AUC	
	DP	SUS-CD	BUSS	Difference	*p*-value	DN	SUS-CD	BUSS	Difference	*p*-value	SUS-CD	BUSS
All patients	191	81 (74, 86)	68 (61, 74)	13 (7, 18)	< 0.001	93	75 (66, 83)	85 (76, 91)	−10 (−17, −3)	0.003	0.81 (0.76, 0.86)	0.82 (0.77, 0.87)
Newly diagnosed	98	85 (76, 90)	67 (58, 76)	17 (9, 26)	< 0.001	35	74 (58, 86)	86 (71, 94)	−11 (−25, 2)	0.046	0.82 (0.74, 0.90)	0.83 (0.75, 0.91)
Suspected relapse	93	76 (67, 84)	69 (59, 77)	8 (1, 14)	0.008	58	76 (63, 85)	84 (73, 92)	−9 (−18, 0)	0.025	0.80 (0.74, 0.87)	0.81 (0.75, 0.88)

Data are *n*, % (95% CI) or *p-*value

Abbreviations: *DP* disease positive, *DN* disease negative, *SUS-CD* simple ultrasound score for Crohn’s disease, *BUSS* bowel ultrasound sonographic score, *sMARIA* simplified magnetic resonance enterography index

The sensitivity of SUS-CD (81% [74, 86]) was significantly greater than that of BUSS (68% [61, 74]) with a difference of 13% (7, 18; *p *< 0.001).

The specificity of SUS-CD (75% [66, 83]) was significantly lower than of BUSS (85% [76, 91]), a difference of −10% (−17, −3; *p *= 0.003).

For the newly diagnosed and suspected relapse group, the sensitivity of SUS-CD was significantly greater than of BUSS, 17% (9, 26; *p *< 0.001) and 8% (1, 14; *p *= 0.008), respectively. There was no significant difference in specificity between SUS-CD and BUSS when stratified by newly diagnosed and suspected relapse.

## Discussion

In the present study, we assessed the diagnostic accuracy of two IUS scoring indices for terminal ileal CD, using prospective data collected as part of a multicentre trial. SUS-CD and BUSS at the previously published thresholds had adequate sensitivity compared to both the histological and magnetic resonance reference standards. Compared to histology, the sensitivity of SUS-CD was significantly greater than that of BUSS. There was no significant difference in specificity. The sensitivity of SUS-CD was also significantly greater than that of BUSS when using the MRE reference standard, but its specificity was significantly lower than of BUSS. The specificity of both indices was numerically higher when the MRE reference standard was adopted.

Like MRE, IUS is an important investigation for CD that influences clinical decision-making [15, 28–30]. Development of IUS activity scores has attracted considerable interest as objective and standardised assessment may increase diagnostic utility across both clinical and research settings [31]. Saevik and co-workers developed SUS-CD in a single-centre prospective study of 40 patients using the simple endoscopic score for CD (SES-CD) as the reference standard [13]. The score was then validated in a dual-centre study, using the same reference standard, in 124 patients with two sonographers performing IUS. Sensitivity for SUS-CD was 95.3% (95% CI 88, 98), specificity 70.3% (56, 82) and ROC AUC 0.92. In our more diverse, multi-institution trial, with numerous sonographers, we found SUS-CD to be sensitive for active TI CD. Specificity was adequate using the MRE reference standard, although lower when compared to histology. Freitas et al tested SUS-CD in a retrospective single-centre study of 50 patients [32]. The reference standard was SES-CD, and a solitary, highly experienced sonographer performed all IUS. They found SUS-CD had an ROC AUC of 0.62 for discriminating between inactive and active CD; sensitivity and specificity were not reported, and the 2 × 2 tables not provided. In their cohort, 40% of their patients had no TI disease. The small, retrospective, single-centre nature of this work limits generalisability.

Allocca et al developed BUSS in a single-centre prospective study of 225 patients, all with an established diagnosis of CD receiving stable treatment, who were undergoing routine assessment [14]. One of two sonographers, with at least 7 years experience, performed IUS. The reference standard was SES-CD. Sensitivity and specificity were 83% (76, 88) and 85% (73, 93), respectively, with a ROC AUC of 0.86 (0.81, 0.91). The same group also assessed BUSS in a prospective study of 49 patients who underwent IUS and colonoscopy before and following treatment with biologics and/or immunosuppressants [20]. These patients all had an established diagnosis of CD for at least 6 months. SES-CD was the reference standard and IUS was again undertaken by two sonographers with at least 7 years experience. BUSS had a sensitivity of 90% (55 to 99) and specificity of 74% (58 to 87) for identifying patients with endoscopic remission. In our more diverse multi-centre, multireader study population, we found BUSS to have adequate sensitivity compared to both reference standards. Specificity was similarly highly compared to the MRE reference standard.

Indeed, in our study, the specificity for both IUS indices was numerically higher when MRE was taken as the reference standard. This may reflect the inherent limitation of a histopathological reference standard which provides superficial sampling of the TI rather than the transmural bowel assessment offered by MRE [18]. Furthermore, in instances of endoscopic skipping, where the luminal surface of the TI is spared because the active inflammation is restricted to intramural portions of the bowel wall, endoscopy may be falsely negative [33, 34]. Interestingly, when compared to the same histological reference, MRE activity scores sMARIA, London score and the ‘extended’ London score also had relatively low specificity of 41%, 64% and 41% respectively [18].

An advantage of histology as a reference standard is that it is independent of the imaging test under consideration. Although MRE is arguably a superior ‘transmural’ reference standard, it shares parameters with IUS. Wall thickness for example is common to both MRE and IUS activity scores, and it is therefore perhaps expected that IUS would fare better against an MRE standard of reference. It would be interesting to fully detail the characteristics of patients who have active disease on IUS (or MRE) but not on histology and vice versa, and how this is influenced by patient cohort (new diagnosis or suspect relapse). Although beyond the scope of the current study, such an analysis is planned.

The development and validation studies for SUS-CD and BUSS occurred in single centres with few sonographers, who were also highly specialised. Indeed, a recent international Delphi consensus panel concluded that it was uncertain if any current IUS scoring systems were appropriate to assess CD activity, highlighting the need for more studies like ours for external validation [31]. The present study is the first to evaluate the performance of these IUS indices in a prospective, multicentre, multireader setting. We considered 284 patients from 8 institutions with 19 operators performing IUS, thus more representative of a real-world setting and likely to reflect expected performance in clinical practice. This is the first study to assess SUS-CD and BUSS against an MRE reference standard, which is important as in the clinical setting, a decision often needs to be made between whether to employ IUS or MRE [2]. Furthermore, MRE is both sensitive and specific for CD providing transmural assessment, and in cases of endoscopic skipping, a reliable alternative to endoscopy [33, 35].

Our work has some limitations. The METRIC trial was completed prior to the development of the SUS-CD and BUSS and so this work is a pragmatic post hoc analysis. In our study, we scored the colour Doppler score component of SUS-CD based on whether the increased flow was focal (less than half the circumference of diseased bowel compared to normal bowel wall of the same patient) or generalised (more than half the circumference of abnormal bowel wall), rather than the exact number of vessels observed (Fig. 2). We also used the original suggested cut-off for active disease. We feel that our methodology is very close to that described for SUS-CD, and that there would have been little to no difference in our estimation of the colour Doppler component. Nevertheless, we cannot exclude that this variation in methodology impacted on results, so future work validating SUS-CD should adopt the exact approach described in the original paper [13]. Notwithstanding, if IUS activity scores are to ultimately be used in routine clinical practice, it is more likely that the colour Doppler parameter will be assessed in a qualitative fashion to save time. This is reflected in recent IUS scores such as the International Bowel Ultrasound Segmental Activity Score (IBUS-SAS) which advocates a qualitative rather than quantitative approach to the assessment of Doppler signal [36]. We did not encounter this issue when deriving BUSS as increased bowel wall flow was a binary score. IUS was performed according to protocol-stipulated parameters, including probe frequency and Doppler flow settings. However, the trial utilised many readers and a range of ultrasound platforms. It is possible that results could have been improved using stricter acquisition protocols, a single manufacturer platform and central reading. However, one of the advantages of the METRIC trial design is that it tested the generalisability of imaging modalities when applied across multiple healthcare settings, reflecting real-world clinical practice [4, 17]. We could not use other reference standards, including ileocolonoscopy scores such as SES-CD to evaluate diagnostic accuracy, as these were not collected as part of METRIC. We were unable to assess inter-observer agreement but this has been studied extensively [8, 37–39]. Reassuringly, De Voogd et al found bowel wall thickness and Doppler flow were reliable, parameters that comprise SUS-CD and BUSS [40]. Our cohort consisted of TI CD exclusively, so we could not evaluate IUS scoring systems for other segments including colonic disease. Our study design did not permit us to consider whether SUS-CD and BUSS are sensitive to treatment response. BUSS has been shown to identify therapeutic response, but more work is needed in this area, especially to evaluate SUS-CD [20]. We could not assess other promising IUS activity scores such as the IBUS-SAS as the data required to calculate these were not collected as part of the METRIC trial [36]. Finally, the METRIC cohort had a relatively high prevalence of active disease given the nature of the recruited patients. Our data is therefore potentially more applicable to patients with higher disease activity, rather than those with more chronic disease and lower levels of enteric inflammation.

## Conclusion

Our study provides real-world evidence that SUS-CD and BUSS are viable IUS indices that are sensitive and specific for active TI CD, especially when compared to an MRE reference standard. More studies like ours in prospective multicentre, multireader settings will facilitate external validation of SUS-CD and BUSS, and establish their suitability for adoption into routine clinical practice.

### Supplementary information

ESM 1 (PDF 467 kb)

ESM 2 (PDF 126 kb)

## Abbreviations

BUSS: Bowel ultrasound score
CD: Crohn’s disease
IUS: Intestinal ultrasound
MRE: Magnetic resonance enterography
sMARIA: Simplified magnetic resonance index of activity
SUS-CD: Simple ultrasound activity score for Crohn’s disease

## Declarations

### Guarantor

The scientific guarantor of this publication is Professor Stuart Taylor.

### Statistics and biometry

One of the authors has significant statistical expertise.

### Study subjects or cohorts overlap

A subset of 111 patients from this study have been reported in Kumar S, Parry T, Mallett S et al (2022) Diagnostic performance of magnetic resonance enterography disease activity indices compared with a histological reference standard for adult terminal ileal Crohn’s disease: experience from the METRIC trial. J Crohns Colitis 2022 ;16(10):1531-1539. doi: 10.1093/ecco-jcc/jjac062. This article evaluated the diagnostic performance of magnetic resonance enterography activity scores.

### Methodology

- prospective
- observational
- multicentre study
