# Supplementary material for: Diagnostic performance of sonographic activity scores for adult terminal ileal Crohn’s disease compared to magnetic resonance and histological reference standards: experience from the METRIC trial
Source: Eur Radiol. 2023 Aug 1;34(1):455–64. doi: 10.1007/s00330-023-09958-6 (PMC10791915; doi:10.1007/s00330-023-09958-6)
Supplement: Supplementary file 1 — (PDF 467 kb) [file 330_2023_9958_MOESM1_ESM.pdf]

METRIC

Site ☐☐☐☐☐☐☐☐☐☐☐☐☐☐ Date of form ☐☐ ☐☐☐ ☐☐☐☐

Patient Number ☐☐☐☐☐ Initials ☐☐☐ Date of birth ☐☐ ☐☐☐ ☐☐☐☐

PATIENT CASE REPORT FORM: US INTERPRETATION

Radiologist Initials______________

USS platform______________________

Are you blinded to other clinical tests and investigations and patient clinical history (other than

previous surgical history and new diagnosis or relapse cohort) ☐ **Yes** ☐ **No**

If N state what information/ test data you are aware of (eg barium FT)_____________________

Scan type (please circle)

Main Metric trial scan Hydosonography substudy Reader agreement substudy

If main Metric scan is this a repeat USS due to unblinded first USS examination? ☐ **Yes** ☐ **No**

**Hydrosonography sub study**			
**Performed using oral contrast given for same day MRE (circle)**	☐ **Yes**		☐ **No**
**Type of oral contrast**		**Volume ingested**	
**Time for ingestion**			

Patient Number ☐☐☐☐☐ Initials ☐☐☐

PATIENT CASE REPORT FORM: US INTERPRETATION: Scan quality

Segment	Quality of segmental visualisation to make correct diagnosis<br><br>1-good/ 2-moderate/ 3-poor/ 4-N/A or excised				If poor visualisation, tick why<br><br>1- overlying bowel gas/ *2- increased patient BM/ 3-Difficult anatomy***/ 4-patient pain/ 5-other (state)*				
	1	2	3	4	1	2	3	4	5
duodenum									
Jejunum*									
ileum									
Terminal ileum**									
Caecum									
Ascending									
Transverse									
Descending									
Sigmoid									
Rectum									

* small bowel from DJ flexure mainly to the left of a diagonal running from the RUQ to LLQ showing typical feathery fold pattern,

** last 10cm of ileum upstream of IV valve/anastomosis

***E.g. low lying caecum

Ileocaecal valve identified ☐ **Yes** ☐ **No**

PATIENT CASE REPORT FORM: US INTERPRETATION Disease Assessment

**Overall disease assessment (to be completed for all patients)**						
	**Normal**		**Equivocal**		**Abnormal**	
**Confidence**	1 (disease definitely not present)	2 (disease probably not present)	3 (disease possibly not present)	4 (disease possibly present)	5 (disease probably present)	6 (disease definitely present)
**Any small bowel disease PRESENT?-** **tick confidence box**						
	1 (disease definitely not active)	2 (disease probably not active)	3 (disease possibly not active)	4 (disease possibly active)	5 (disease probably active)	6 (disease definitely active)
**If present (confidence score ≥3) is it ACTIVE? tick confidence box**						
	1 (disease definitely not present)	2 (disease probably not present)	3 (disease possibly not present)	4 (disease possibly present)	5 (disease probably present)	6 (disease definitely present)
**Any colonic disease PRESENT?** **tick confidence box**						
	1 (disease definitely not active)	2 (disease probably not active)	3 (disease possibly not active)	4 (disease possibly active)	5 (disease probably active)	6 (disease definitely active)
**If present (confidence score ≥3) is it ACTIVE? tick confidence box**						

PATIENT CASE REPORT FORM: US INTERPRETATION Other findings

**Lymphadenopathy (0-3)**	☐ **0**	☐ **1**	☐ **2**	☐ **3**
**Abnormal free fluid (Y/N)**	☐ **Yes**		☐ **No**	
**Abscess present**	☐ **Yes**		☐ **No**	
If yes please state size & location				
**Fistula present (circle all that apply)**	☐ **Yes**		☐ **No**	
If yes please circle location		Ileo-ilealIleo-colic entero-cutaneous ileo-vesical<br>colon-vesicaljejuno- jejunaljejuno-colic<br>Other (state______________________________)		
**Other small bowel diagnosis (e.g. adhesions, meckels, radiation enteritis etc**	☐ **Yes**		☐ **No**	
**If yes please state**				
**Extra enteric findings (e.g. aortic aneurysm gallstones, solid organ abnormality, phelgmon)**	☐ **Yes**		☐ **No**	
If yes please state				
**Are you recommending any further tests?**	☐ **Yes**		☐ **No**	
If yes please state which				

METRIC

Patient Number | | | | | | Initials | | | |

PATIENT CASE REPORT FORM: US INTERPRETATION Disease Presence

Please complete for each segment	Confidence of disease PRESENCE					
	**Normal**		**Equivocal**		**Abnormal**	
**Segment**	1 (disease definitely not present)	2 (disease probably not present)	3 (disease possibly not present)	4 (disease possibly present)	5 (disease probably present)	6 (disease definitely present)
**Duodenum (D)**						
**Jejunum (J)**						
**Ileum (I)**						
**Terminal ileum (TI)***						
**Caecum (C)**						
**Ascending colon (A)**						
**Transverse colon (T)**						
**Descending colon (D)**						
**Sigmoid (S)**						
**Rectum (R)**						

*throughout, if TI disease is contiguous for over 10cm count just as TI not TI and ileum

PATIENT CASE REPORT FORM: US INTERPRETATION Disease Activity

Confidence of disease ACTIVITY Please complete for each segmentif confidence scores 3-6 for disease presence above i.e. **present or equivocal**

		Normal		Equivocal		Active	
**Segment**	No disease (i.e. confidence scores 1 or 2 for disease presence)	1 (disease definitely not active)	2 (disease probably not active)	3 (disease possibly not active)	4 (disease possibly active)	5 (disease probably active)	6 (disease definitely active)
**Duodenum (D)**							
**Jejunum (J)**							
**Ileum (I)**							
**Terminal ileum (TI)**							
**Caecum (C)**							
**Ascending colon (A)**							
**Transverse colon (T)**							
**Descending colon (D)**							
**Sigmoid (S)**							
**Rectum (R)**							

METRIC

Patient Number ☐☐☐☐☐ Initials ☐☐☐

PATIENT CASE REPORT FORM: US INTERPRETATION Disease Description Disease Site 1

PLEASE COMPLETE FOR EACH DISEASE SITE (DEFINED AS >3CM OF NORMAL BOWEL BETWEEN DISEASE SITES). USE ONE TABLE FOR EACH DISEASE SITE. ONLY RECORD SEGMENTS WHICH IF YOU HAVE A CONFIDENCE SCORE OF 3 OR MORE FOR DISEASE PRESENCE. USE SCORE DEFINITIONS AT THE START OF THIS CRF

Complete for equivocal or abnormal sites (i.e. confidence scores 3-6). Complete additional tables as required if multiple disease sites per segment

Location	Tick one location	Single Wall thickness (mm) thickest portion	Wall thickening	Length of abnormal bowel (cm)	Stensois causing functional obstruction	Mesenteric fat echogenicity	Anti-mesenteric border	Mesenteric border	Submucosal layer	Submucosal layer echogenicity	Submucosal layer clarity	Mucosal layer	Ulceration	Doppler vascular pattern axial section	Peristaltic distension	Does the segment contain established fibrosis (Y/N)	Segmental disease severity assessment	Segment shows active disease (Y/N)
**Duo**																		
**J**																		
**I**																		
**TI**																		
**C**																		
**A**																		
**Des**																		
**S**																		
**R**																		

Patient Number | | | | | | Initials | | | |

PATIENT CASE REPORT FORM: US INTERPRETATION Disease Description Disease Site 2
**PLEASE COMPLETE FOR EACH DISEASE SITE (DEFINED AS >3CM OF NORMAL BOWEL BETWEEN DISEASE SITES). USE ONE TABLE FOR EACH DISEASE SITE. ONLY RECORD SEGMENTS WHICH IF YOU HAVE A CONFIDENCE SCORE OF 3 OR MORE FOR DISEASE PRESENCE. USE SCORE DEFINITIONS AT THE START OF THIS CRF**
**Complete for equivocal or abnormal sites (i.e. confidence scores 3-6). Complete additional tables as required if multiple disease sites per segment**

Location	Tick one location	Single Wall thickness (mm) thickest portion	Wall thickening	Length of abnormal bowel (cm)	Stensois causing functional obstruction	Mesenteric fat echogenicity	Anti-mesenteric border	Mesenteric border	Submucosal layer	Submucosal layer echogenicity	Submucosal layer clarity	Mucosal layer	Ulceration	Doppler vascular pattern axial section	Peristaltic distension	Does the segment contain established fibrosis (Y/N)	Segmental disease severity assessment	Segment shows active disease (Y/N)
**Duo**																		
**J**																		
**I**																		
**TI**																		
**C**																		
**A**																		
**Des**																		
**S**																		
**R**																		

PATIENT CASE REPORT FORM: US INTERPRETATION Disease Description Disease Site 3

PLEASE COMPLETE FOR EACH DISEASE SITE (DEFINED AS >3CM OF NORMAL BOWEL BETWEEN DISEASE SITES). USE ONE TABLE FOR EACH DISEASE SITE. ONLY RECORD SEGMENTS WHICH IF YOU HAVE A CONFIDENCE SCORE OF 3 OR MORE FOR DISEASE PRESENCE. USE SCORE DEFINITIONS AT THE START OF THIS CRF

Complete for equivocal or abnormal sites (i.e. confidence scores 3-6). Complete additional tables as required if multiple disease sites per segment

Location	Tick one location	Single Wall thickness (mm) thickest portion	Wall thickening	Length of abnormal bowel (cm)	Stensois causing functional obstruction	Mesenteric fat echogenicity	Anti-mesenteric border	Mesenteric border	Submucosal layer	Submucosal layer echogenicity	Submucosal layer clarity	Mucosal layer	Ulceration	Doppler vascular pattern axial section	Peristaltic distension	Does the segment contain established fibrosis (Y/N)	Segmental disease severity assessment	Segment shows active disease (Y/N)
Duo																		
J																		
I																		
TI																		
C																		
A																		
Des																		
S																		
R																		

Patient Number ☐☐☐☐☐ Initials ☐☐☐

PATIENT CASE REPORT FORM: US INTERPRETATION Disease Description Disease Site 4
PLEASE COMPLETE FOR EACH DISEASE SITE (DEFINED AS >3CM OF NORMAL BOWEL BETWEEN DISEASE SITES). USE ONE TABLE FOR EACH DISEASE SITE. ONLY RECORD SEGMENTS WHICH IF YOU HAVE A CONFIDENCE SCORE OF 3 OR MORE FOR DISEASE PRESENCE. USE SCORE DEFINITIONS AT THE START OF THIS CRF
Complete for equivocal or abnormal sites (i.e. confidence scores 3-6). Complete additional tables as required if multiple disease sites per segment

Location	Tick one location	Single Wall thickness (mm) thickest portion	Wall thickening	Length of abnormal bowel (cm)	Stensois causing functional obstruction	Mesenteric fat echogenicity	Anti-mesenteric border	Mesenteric border	Submucosal layer	Submucosal layer echogenicity	Submucosal layer clarity	Mucosal layer	Ulceration	Doppler vascular pattern axial section	Peristaltic distension	Does the segment contain established fibrosis (Y/N)	Segmental disease severity assessment	Segment shows active disease (Y/N)
Duo																		
J																		
I																		
TI																		
C																		
A																		
Des																		
S																		
R																		

METRIC

Patient Number | | | | | | Initials | | | |

PATIENT CASE REPORT FORM: US INTERPRETATION Disease Description Disease Site 5																		
PLEASE COMPLETE FOR EACH DISEASE SITE (DEFINED AS >3CM OF NORMAL BOWEL BETWEEN DISEASE SITES). USE <u>ONE</u> TABLE FOR EACH DISEASE SITE. ONLY RECORD SEGMENTS WHICH IF YOU HAVE A CONFIDENCE SCORE OF 3 OR MORE FOR DISEASE PRESENCE. USE SCORE DEFINITIONS AT THE START OF THIS CRF																		
Complete for equivocal or abnormal sites (i.e. confidence scores 3-6). Complete additional tables as required if multiple disease sites per segment																		
Location	Tick one location	Single Wall thickness (mm) thickest portion	Wall thickening	Length of abnormal bowel (cm)	Stensois causing functional obstruction	Mesenteric fat echogenicity	Anti-mesenteric border	Mesenteric border	Submucosal layer	Submucosal layer echogenicity	Submucosal layer clarity	Mucosal layer	Ulceration	Doppler vascular pattern axial section	Peristaltic distension	Does the segment contain established fibrosis (Y/N)	Segmental disease severity assessment	Segment shows active disease (Y/N)
Duo																		
J																		
I																		
TI																		
C																		
A																		
Des																		
S																		
R																		

PATIENT CASE REPORT FORM: US INTERPRETATION Disease Description Disease Site 6																		
PLEASE COMPLETE FOR EACH DISEASE SITE (DEFINED AS >3CM OF NORMAL BOWEL BETWEEN DISEASE SITES). USE ONE TABLE FOR EACH DISEASE SITE. ONLY RECORD SEGMENTS WHICH IF YOU HAVE A CONFIDENCE SCORE OF 3 OR MORE FOR DISEASE PRESENCE. USE SCORE DEFINITIONS AT THE START OF THIS CRF																		
Complete for equivocal or abnormal sites (i.e. confidence scores 3-6). Complete additional tables as required if multiple disease sites per segment																		
Location	Tick one location	Single Wall thickness (mm) thickest portion	Wall thickening	Length of abnormal bowel (cm)	Stensois causing functional obstruction	Mesenteric fat echogenicity	Anti-mesenteric border	Mesenteric border	Submucosal layer	Submucosal layer echogenicity	Submucosal layer clarity	Mucosal layer	Ulceration	Doppler vascular pattern axial section	Peristaltic distension	Does the segment contain established fibrosis (Y/N)	Segmental disease severity assessment	Segment shows active disease (Y/N)
Duo																		
J																		
I																		
TI																		
C																		
A																		
Des																		
S																		
R																		

Completed by:

Print name: *Signature:* *Date:*

Office Use Only:

Received by (Print name & sign): *Date:* *Entered by: (Print name & sign):* *Date:*
