# Supplementary material for: Diagnostic performance of sonographic activity scores for adult terminal ileal Crohn’s disease compared to magnetic resonance and histological reference standards: experience from the METRIC trial
Source: Eur Radiol. 2023 Aug 1;34(1):455–64. doi: 10.1007/s00330-023-09958-6 (PMC10791915; doi:10.1007/s00330-023-09958-6)
Supplement: Supplementary file 2 — (PDF 126 kb) [file 330_2023_9958_MOESM2_ESM.pdf]

Diagnostic performance of sonographic activity scores for adult terminal ileal Crohn's disease compared to magnetic resonance and histological reference standards: experience from the METRIC trial

Electronic Supplementary Material

Appendices

Appendix 1. Standardised clinical report form (CRF) documenting conventional IUS observations.

Appendix 2. Definitions of the SUS-CD and BUSS

SUS-CD = SUS-CD = bowel wall thickness + colour Doppler score

Score	0	1	2	3
**Bowel wall thickness**	<3mm	3-4.9mm	5-7.9mm	≥8mm
**Colour Doppler signal**	No or single vessel per cm^2	2-5 vessels per cm^2	>5 vessels per cm^2	

Thus, the score for each segment ranges from 0 to 5.

BUSS = 0.75 x bowel wall thickness + 1.65 x bowel wall flow

Bowel wall thickness in mm

Bowel wall flow – defined as (0) absence or (1) presence of vascular signals at colour Doppler

Appendix 3. Definition of the sMARIA.

sMARIA = (1 x wall thickness > 3 mm) + (1 x wall oedema) + (1 x fat stranding) + (2 x ulcers)

Wall thickness >3mm scores 1 point

Presence of oedema scores 1 point

Presence of fat stranding scores 1 point (we used perimural stranding as a surrogate for this)

Presence of ulcers scores 2 points

Thus, the score for each segment ranges from 0 to 5.

Appendix 4. Contingency table of HAI versus sMARIA.

		**sMARIA**		**Total**
		Active	Inactive	
**HAI**	Active	74	15	89
	Inactive	13	9	22
	Missing	104	69	173
**Total**		191	93	284

Supplemental Table 1. Histological Activity Index (HAI)

**Inflammatory Activity**	**Score**	**Histopathologic Defining Characteristics**
Inactive/ Quiescent/ Normal	0	No epithelial infiltration by neutrophils.
Mildly Active	1	Neutrophil infiltration of <50% of sampled crypts or cross-sections, no ulcers, or erosions.
Moderately Active	2	Neutrophil infiltration of ≥ 50% of sampled crypts or cross-sections, no ulcers, or erosions.
Severely Active	3	Erosion or ulceration, irrespective of other features.

a. Inactive colitis with no cryptitis or crypt abscesses; HAI=0.

b. Mildly active colitis with one crypt abscess (arrow); HAI=1

c. Moderately active colitis with cryptitis involving >50% of crypts (arrows); HAI=2.

d. Severely active colitis with ulceration; HAI=3.

Supplemental Table 2. Patient demographics and clinical characteristics of those with available terminal ileal biopsies (n = 111).

Characteristic	Newly diagnosed n=75	Suspected relapse n=36	All patients n=111
**Age (years)**	26 (20, 39)	34 (26, 42)	28 (21, 40)
**Sex**			
Male	33 (44)	23 (64)	55 (50)
Female	42 (56)	13 (36)	56 (50)
**Medication**			
ASA	10 (10)	6 (9)	16 (10)
Anti-TNF antibodies	3 (3)	2 (3)	5 (3)
Immunomodulator	7 (7)	17 (27)	24 (15)
Steroid	29 (30)	12 (19)	41 (26)
**HBI**	3.5 (1, 6)	4.5 (2, 9)	4 (2, 6)
**EQ-5D**	70 (50, 83)	65 (40, 80)	70 (50, 80)
**CRP (mg/L)**	11.2 (2.9, 28.5)	6.2 (1.8, 22.8)	8.5 (2.5, 25.8)
**Calprotectin (μg/g)**	466 (137, 686)	405 (247, 780)	437 (151, 716)
**History of bowel surgery**	7 (9)	14 (39)	21 (19)
**US platform**			
GE Healthcare	19 (25)	7 (19)	26 (23)
Philips	11 (15)	3 (8)	14 (13)
Siemens	19 (25)	14 (39)	33 (30)
Toshiba	26 (35)	12 (33)	38 (34)

All data are n (%) or median (inter-quartile range).

Abbreviations: ASA = acetylsalicylic acid, TI = terminal ileal, TNF = tumour necrosis factor, HBI = Harvey-Bradshaw Index, EQ-5D = EuroQol five-dimension questionnaire, CRP = C-reactive protein, US = ultrasound.

Missing data: HBI = 9, EQ-5D = 12, CRP = 10, Calprotectin = 50.
